# In Vitro/In Silico Potential of High-Yield Essential Oils for Management of Postharvest Fungi

**DOI:** 10.3390/metabo16040239

**Published:** 2026-03-31

**Authors:** José Manuel Pineda-Ríos, Danae Abigail Ruiz-Aguilar, Óscar Morales-Galván, Ma. de Lourdes Catalina Arévalo-Galarza, Rosa María López-Romero, Victoria Ayala-Escobar, Monserrat Vázquez-Sánchez, Luis Francisco Salomé-Abarca

**Affiliations:** 1 Departamento de Parasitología Agrícola, Universidad Autónoma Chapingo, Km 38.5 Carretera Federal México-Texcoco, Texcoco de Mora 56230, Estado de México, Mexico; jpinedar@chapingo.mx (J.M.P.-R.); dana.ruiz.aguilar@gmail.com (D.A.R.-A.); omoralesg@chapingo.mx (Ó.M.-G.); 2 Posgrado en Recursos Genéticos y Productividad-Fruticultura, Colegio de Postgraduados, Campus Montecillo, Km 36.5 Carretera México-Texcoco, Montecillo, Texcoco de Mora 56230, Estado de México, Mexico; larevalo@colpos.mx (M.d.L.C.A.-G.); rosal@colpos.mx (R.M.L.-R.); ayalav@colpos.mx (V.A.-E.); vazquez.monserrat@colpos.mx (M.V.-S.)

**Keywords:** bioactivity prediction, multivariate selection, volatile formulation, cutinase

## Abstract

**Background/Objectives**: Microbial infections represent a major challenge in the food processing chain. Postharvest fungal control has historically relied on chemical control; however, their use is increasingly restricted due to environmental and health risks. Therefore, the aim of this study was to evaluate the antifungal potential of essential oils obtained from high-yield plant species and characterize the potential mechanisms of action of their major volatiles, with the goal of proposing a prospective formulation for the control of postharvest fungi. **Methods**: Cinnamon, rosemary, allspice, and Peruvian pepper essential oils were extracted by hydrodistillation, tested against *Botrytis cinerea* and *Colletotrichum* sp., and analyzed by gas chromatography-mass spectrometry. Finally, in silico bioactivity analyses were performed on the most abundant volatiles. **Results**: Cinnamon and rosemary produced the most effective oils against both fungal species. Cinnamaldehyde, cinnamyl acetate, eugenol, methyleugenol, (+)-2-bornanone, eucalyptol, *α*-phellandrene, and *β*-myrcene were some of the most abundant volatiles in the analyzed oils. In silico analyses predicted 56 antifungal mechanisms, including inhibition of cell membrane and wall synthesis, affectation of primary metabolism, inhibition of molecular processes, redox homeostasis, and protein degradation and cutinase inhibition. The last one is a specific mechanism mediating in vivo plant-fungal interactions found exclusively in *β*-terpinene and *β*-ocimene. **Conclusions**: Compounds with cutinase inhibition activity such as *β*-terpinene and *β*-ocimene are of great potential to complement the activity of other bioactive compounds. According to literature and in silico analyses the mixture of cinnamaldehyde, eugenol, *β*-terpinene and *β*-ocimene could be a potential formulation for the management of postharvest fungi.

## 1. Introduction

Agricultural production faces multiple challenges that negatively affect both food yield and quality. For instance, approximately 15% of fruit and vegetables are lost during postharvest handling and management. Product losses occur when they are not consumed due to quality deterioration, which may result from misshapen fruits, mechanical damage, or pest infestations associated with inadequate storage conditions [1]. Consequently, maintaining appropriate storage conditions remains a persistent challenge for the food industry. It is estimated that 40 to 50% of stored products are lost during storage, generating an annual economic loss of approximately USD 750 billion related to food preservation efforts [2].

Among postharvest challenges, microbial infections represent a major concern because they may arise at any stage of the food supply chain. Disease symptoms might appear during sorting, packaging, transportation, storage, or even at the point of consumption [3]. Postharvest diseases are mainly caused by fungi, where *Botrytis cinerea* and *Colletotrichum* spp. are the two most frequent and destructive species. Gray mold, caused by *B. cinerea*, is one of the most relevant postharvest diseases affecting fruits, vegetables, and ornamental crops. *B. cinerea* is responsible for substantial economic losses, estimated to be between USD 10 and 100 billion annually, and can affect 20–60% of stored products depending on the commodity and storing technology employed [4]. Similarly, anthracnose caused by *Colletotrichum* spp. leads to considerable losses during both the pre- and postharvest stages of crop production. *B. cinerea* and *Colletotrichum* sp. negatively affect the quality and productivity of several crops, with the severity of infection largely influenced by host species and the environmental conditions. In severe infections, anthracnose can result in 100% production losses [5].

The control of fungal diseases during the postharvest handling of fruits and vegetables has historically relied on the application of synthetic chemical fungicides. However, the use of such chemicals is increasingly restricted due to concerns regarding their residual persistence, environmental pollution, potential risk to consumer health, and the development of fungal resistance to conventional fungicides [6]. Consequently, antimicrobial agents derived from natural products have attracted growing scientific attention as potential alternatives for postharvest diseases management [7]. Among these alternatives, essential oils have been widely investigated for agricultural pest management applications. Their potential as substitutes for conventional pesticides is largely associated with their physicochemical features, including high volatility and natural origin, which reduce the risk of harmful residue accumulation in agricultural products and the environment [8].

Despite their potential, several functional limitations must be considered for the effective development of essential oil-based antifungal agents. Most studies have evaluated essential oils as contact agents incorporated into culture media under controlled laboratory conditions [9]. However, the direct application of oily substances onto plant or fruit surfaces may cause tissue burns. In addition, several plant essential oils contain volatile metabolites responsible for intense odors and flavors that may negatively affect the organoleptic quality of treated food products. To mitigate these drawbacks, essential oils with sweet odor notes have been evaluated in vapor-phase applications against phytopathogenic fungi, showing promising antifungal activity [10]. In this context, vapor-phase applications are attractive because they employ the volatility of essential oil, exerting antifungal effects without direct contact with fruit or plant tissues.

Another limiting factor for the large-scale application of essential oil as antifungal agents is their relatively low extraction yield, less than 0.1%, in several plant species [11,12]. Due to low yield, substantial amounts of plant biomass are required to meet industrial demands. Furthermore, the chemical composition of essential oil can vary considerably depending on agronomic practices, geographic origin, and environmental conditions, potentially leading to variations in their biological activity [13]. Therefore, the identification of high-essential-oil-yielding plant species, with essential oil yields higher than 0.5% [14], the characterization of the antifungal activity of their major metabolites, and the use of pure compounds are quintessential steps to be considered for the design of essential oil-based antifungal agents. For instance, formulations made of pure compounds could help to easily correlate variations in antifungal activity with changes in the molar ratios of compounds within the mixtures. Therefore, understanding the antifungal properties of individual metabolites and their interactions in combined formulations may reduce the reliance on whole essential oils, facilitating the development of more standardized and reproducible antifungal formulations. In this context, the aim of this study was to evaluate the antifungal potential of essential oils obtained from high-yield plant species and to characterize the potential mechanisms of action of their major chemical constituents, with the goal of proposing a prospective volatile formulation for the control of postharvest fungi.

## 2. Materials and Methods

### 2.1. Plant Material

The plant material consisted of commercial bark of cinnamon (*Cinnamomum verum* J. Presl (Lauraceae)) obtained from the cooperative “La Joya del Campo SC de RL”, located in Zozocolco, Veracruz, Mexico. Fruits of allspice (*Pimenta dioica* L. (Myrtaceae)) were commercially sourced from Texcapa Oils^®^, located in Tilzapota, Atzalan, Veracruz, Mexico. Mature fruits of the Peruvian pepper (*Schinus molle* L. (Anacardiaceae)) and rosemary leaves (*Salvia rosmarinus* Schleid. (Lamiaceae)) were collected at the Colegio de Postgraduados, Campus Montecillo, located in Texcoco de Mora, Estado de Mexico, Mexico. Cinnamon and allspice were sun-dried; Peruvian pepper fruits were dried for 24 h at 50 °C in an oven. Rosemary was used fresh. The identification of herbarium specimens was based on morphological characteristics, using dichotomous keys and comparisons with specimens deposited in the CHAPA herbarium at the Colegio de Postgraduados and digitalized specimens in the Northwestern Herbarium Network. Identities were verified by Dr. Monserrat Vázquez Sánchez, who belong to the Botany Graduate Program at the Colegio de Postgraduados. Scientific names were confirmed using the Tropicos, International Plant Name Index (IPNI), and Plants of the World Online (POWO) databases.

### 2.2. Hydrodistillation

To obtain essential oils, cinnamon, allspice, and Peruvian pepper fruits were ground in a blender until a fine powder was obtained. Rosemary leaves were cut into small pieces (smaller than 0.5 mm). Each material was extracted individually by hydrodistillation for 3 h using 250 g of plant material. The essential oil was collected using a Clevenger-type system and recovered from the top phase of the trap. The oil was dried over anhydrous sodium sulfate and then weighed to calculate the extraction yield. The essential oil yield was calculated by multiplying the oil weight by 100 and dividing it by the initial sample weight (250 g). Four replicates were performed for each plant species, and results were expressed as percent yield (%) ± standard error.

### 2.3. Gas Chromatography-Mass Spectrometry Analysis (GC-MS)

Essential oils were analyzed using a gas chromatograph (GC) Agilent Technologies (Santa Clara, CA, USA) (7890A) coupled with a single quadrupole mass spectrometer (MS) Agilent Technologies (Santa Clara, CA, USA) (5975C). The GC-MS system was equipped with an HP-5MS column (30 m × 0.250 mm internal diameter and 0.25 µm film thickness, J&W Science, Folsom, CA, USA) for volatile separation. Helium (99.999% purity) was used as the carrier gas with a flow rate of 1 mL/min. One microliter of essential oil was diluted in 1 mL of hexane, and from this dilution, 1 µL was injected for chromatographic analysis.

For cinnamon, allspice, and Peruvian pepper oils, the oven was initially set at 50 °C for 1 min, then increased by 4 °C/min up to 100 °C and held for 1 min, followed by a ramp of 7 °C/min up to 180 °C, held for 1 min, and then increased by 20 °C/min up to 240 °C, held again for 1 min. For rosemary oil, the oven started at 50 °C for 1 min, increased by 4 °C/min up to 80 °C and held for 1 min, then ramped up at 2 °C/min to 93 °C and held for 1 min, increased to 180 °C at 7 °C/min (held 1 min), and finally ramped to 240 °C at 25 °C/min, with a final 1 min hold. The injector port temperature was 240 °C for all analyses on Split mode (10:1). The ion source and quadrupole temperatures were 230 °C and 150 °C, respectively. The transfer line was set to 280 °C. Ionization energy in electronic impact (EI)-mode was 70 eV, and mass data were acquired in SCAN mode (30–550 *m*/*z*). Compound identification was carried out by comparing the mass spectra and/or their retention times with those in the NIST library (v.2014) and those of commercial standards.

(+)-2-Bornannone (W223018), 3-Carene (115576), *α*-Phellandrene (W285611), *α*-Pinene (147524), α-Terpineol (CRM40428), *β*-Myrcene (M-0382), *β*-Pinene (W290315), *γ*-Terpinene (223190), Camphene (456055), Cinnamyl acetate (166170), D-Limonene (255270), (*E*)-*β*-Caryophyllene (C-9653), (*E*)-Cinnamaldehyde (C80685), Eucalyptol (C80601), Eugenol (E51791), *o*-Cymene (255270), Ocimene mixture (W353901), Metyleugenol (284424), and Benzaldehyde (B1334) were obtained from Sigma-Aldrich (Toluca, Edo. Mex., MEX/ St. Louis, MO, USA); *trans*-*β*-Ocimene (A0007408) was obtained from Chemos GmbH (Altdorf, BY, DEU).

### 2.4. Microorganisms

Strains of *Botrytis cinerea* and *Colletotrichum* sp., respectively isolated from strawberry and mango, were used for antifungal tests. The strains belong to the fungal collection of the Postharvest Disease Laboratory of the Plant Pathology Graduate Program at the Colegio de Postgraduados.

### 2.5. Antifungal Activity Tests

An 8 mm diameter plug of *B. cinerea* or *Colletotrichum* sp. mycelium was placed at the center of a potato dextrose agar (PDA)-Petri dish. The mycelium was taken from the young edge of fungal colonies, 3 and 8 days old for *Botrytis* and *Colletotrichum*, respectively. Four concentrations of each essential oil were tested: 1000, 750, 500, and 250 mg L^−1^ per Petri dish volume. To achieve the required concentrations, the oil was diluted with glycerol. As negative controls, mixtures of sterile water and glycerol were used at the same concentrations as the essential oils. The essential oils were poured on sterile filter paper disks placed on the lid of the Petri dish. The culture dishes were sealed with parafilm and incubated upside-down (28 °C) to avoid direct contact of essential oil with the culture medium or the fungal mycelium. The mycelial growth was monitored every 24 h for 5 days. Four replicates were performed for each treatment, with each Petri dish representing an experimental unit, totaling 160 experimental units (80 per fungal genus).

### 2.6. In Silico Analysis of Potential Antifungal Mechanisms of Volatile Compounds

The compounds with the highest chromatographic abundance from each essential oil were analyzed using the online Prediction of Activity Spectra for Substances (PASS)-software (v.2.0) to predict potential mechanisms associated with their antifungal activity [15]. For the prediction of mechanisms, the Simplified Molecular Input Line Entry System (SMILES)-descriptors of each identified compound were inputted into the software. The numerical values for each compound represent the difference between the predicted probability of being active (Pa) and being inactive (Pi). Only mechanisms with moderate potential (0.3–0.5) and high bioactive potential (>0.5) were reported [16].

### 2.7. Data Analysis

The essential oil yields were subjected to tests for normality and homogeneity of variance. Tukey’s mean comparison test (α = 0.05) was subsequently performed to determine statistical differences among essential oils- yields. Data from in silico analysis were evaluated using hierarchical cluster analysis (HCA) which was projected on a Bi-plot constructed from a principal component analysis (PCA), allowing the observation of similarities between compounds according to their predicted potential anti-fungal mechanisms of action. The data were scaled using the unit variance (UV) method.

## 3. Results

### 3.1. Essential Oil Yield

**Figure 1:**
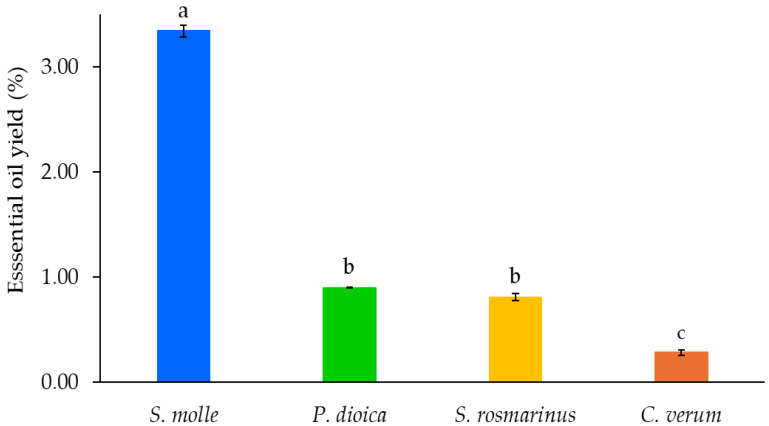
Essential yield comparison between four plant species, *Schinus molle*, *Pimienta dioica*, *Salvia rosmarinus* and *Cinnamomum verum*. Data represents average values (n = 4) ± standard error. Different letters above each bar indicates significant differences in a Tukey test (α = 0.05).

The analysis of essential oil yields revealed significant differences among the four plant materials (*p* ≤ 0.05). Peruvian pepper fruits exhibited the highest essential oil yield, followed by allspice and rosemary, which were not significantly different between each other (*p* > 0.05). Cinnamon bark produced the lowest yield among the species evaluated (*p* ≤ 0.05). The average yields for Peruvian pepper, allspice, rosemary, and cinnamon were 3.34%, 0.90%, 0.81%, and 0.28%, respectively. Thus, Peruvian pepper fruits produced approximately five times more essential oil than allspice and rosemary, and nearly twelve times more than cinnamon (Figure 1). The results highlighted the markedly higher essential oil yield of Peruvian pepper compared with the other evaluated plant materials.

### 3.2. Antifungal Activity

**Figure 2:**
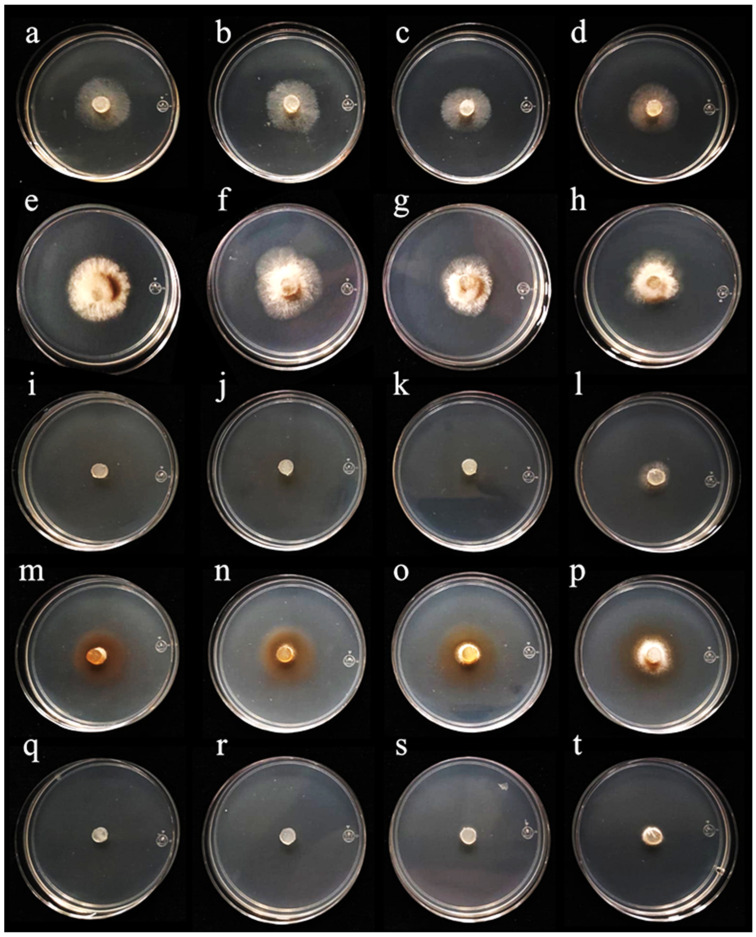
Antifungal effects of four essential oils against *Botrytis cinerea*. (**a**–**d**) represent negative controls. (**e**–**h**) represent 1000 mg/L, 750 mg/L, 500 mg/L, and 250 mg/L of *Schinus molle* essential oil, respectively. (**i**–**l**) represent 1000 mg/L, 750 mg/L, 500 mg/L, and 250 mg/L *Cinnamomun verum* essential oil, respectively. (**m**–**p**) represent 1000 mg/L, 750 mg/L, 500 mg/L, and 250 mg/L of *Pimienta dioica* essential oil, respectively. (**q**–**t**) represent 1000 mg/L, 750 mg/L, 500 mg/L, and 250 mg/L of *Salvia rosmarinus* essential oil, respectively. Pictures were taken at 5 days of growth.

**Figure 3:**
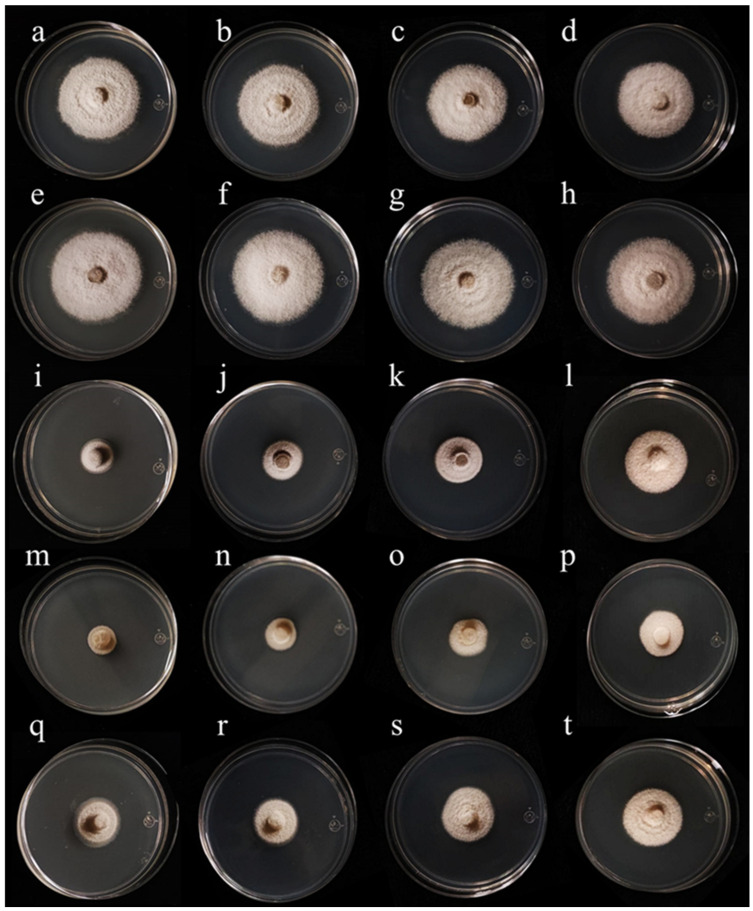
Antifungal effects of four essential oils against *Colletotrichum* spp. (**a**–**d**) represent negative controls. (**e**–**h**) represent 1000 mg/L, 750 mg/L, 500 mg/L, and 250 mg/L of *Schinus molle* essential oil, respectively. (**i**–**l**) represent 1000 mg/L, 750 mg/L, 500 mg/L, and 250 mg/L *Cinnamomun verum* essential oil, respectively. (**m**–**p**) represent 1000 mg/L, 750 mg/L, 500 mg/L, and 250 mg/L 1 of *Pimienta dioica* essential oil, respectively. (**q**–**t**) represent 1000 mg/L, 750 mg/L, 500 mg/L, and 250 mg/L of *Salvia rosmarinus* essential oil, respectively. Pictures were taken at 5 days of growth.

Independently of the essential oil yield of plant species, their antifungal activity degree represents a critical factor for their potential agronomical application. Therefore, the antifungal effects of the four essential oils were tested against *B. cinerea* and *Colletotrichum* sp. The results showed that, despite its high essential oil yield, Peruvian pepper did not produce inhibitory activity against either *B. cinerea* (Figure 2) or *Colletotrichum* sp. (Figure 3). In contrast, essential oils from cinnamon, allspice, and rosemary inhibited both fungal species, with a more pronounced effect against *B. cinerea*. In all tests, the degree of fungal inhibition was dependent on both essential oil concentration and exposure time. Therefore, antifungal activity is not necessarily correlated with the essential oil yield.

Notably, although allspice essential oil reduced the growth of *B. cinerea*, it also induced the secretion of pigments into the culture medium (Figure 2m–p). The production of these pigments strongly suggests the synthesis of specialized metabolites that could include toxins. Therefore, the use of allspice essential oil to manage *B. cinerea* might be limited. In contrast, cinnamon and rosemary essential oils showed strong inhibitory activity against this *B. cinerea*. Regarding *Colletotrichum* sp., allspice essential oil showed a clear inhibitory activity without inducing visible pigment secretion into the culture medium. Similarly to the observations for *B. cinerea*, Peruvian pepper essential oil did not show inhibitory effects against *Colletotrichum* sp., whereas cinnamon and rosemary essential oils produced the strongest inhibitory effects against this phytopathogen (Figure 3).

**Figure 4:**
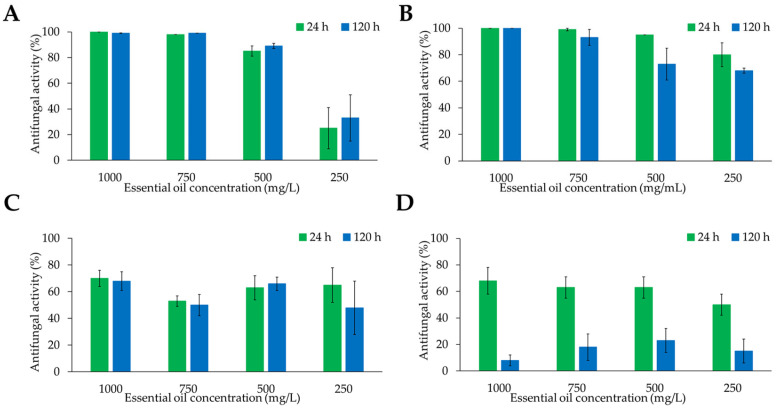
Antifungal activity of essential oils obtained from (**A**) *Cinnamomum verum*, (**B**) *Salvia rosmarinus*, (**C**) *Pimenta dioica*, and (**D**) *Schinus molle* against *Botrytis cinerea* after 24 y 120 h of growth.

The strongest antifungal effects of the evaluated essential oils against both fungal species were observed at the early (24 h) and final (120 h) time points of the experiment. For *B. cinerea*, completely inhibition of mycelial growth at 24 h was achieved only by rosemary and cinnamon essential oils. Moreover, their antifungal effects remained almost unchanged after 120 h at concentrations of 1000 and 750 mg/L. For cinnamon essential oil, the antifungal activity at 500 mg/L decreased by only 10% after 120 h. However, at a lower concentration of 250 mg/L, the inhibitory effect was reduced approximately by 80% at the same time point (Figure 4A). Similarly, rosemary essential at 1000 and 750 mg/L completely inhibited the growth of *B. cinerea* at 24 h during throughout the 120 h evaluation period (Figure 4B). Interestingly, rosemary essential oil showed a reduction of 20% of bioactivity at 500 mg/L after 120 h. Despite this decrease, the inhibitory effect of rosemary oil remained higher than that determined for cinnamon essential oil at the same concentration (Figure 4B). In contrast, allspice essential oil reduced the growth of *B. cinera* approximately 50–70% across all tested concentrations. Peruvian pepper essential oil showed a similar range of inhibitory activity at 24 h; however, its antifungal activity declined markedly over time, losing approximately 75% of its inhibitory effects after 120 h (Figure 4C,D). Overall, rosemary essential oil exhibited the most consistent and sustained antifungal activity against *B. cinerea*.

**Figure 5:**
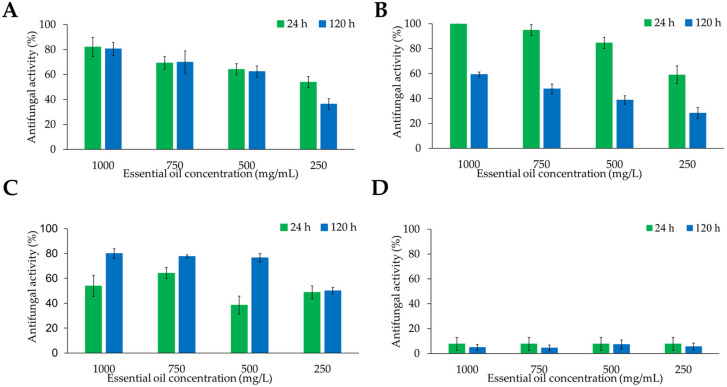
Antifungal activity of essential oils obtained from (**A**) *Cinnamomum verum*, (**B**) *Salvia rosmarinus*, (**C**) *Pimenta dioica*, and (**D**) *Schinus molle* against *Colletotrichum* spp. after 24 y 120 h of growth.

Regarding the antifungal activity against *Colletotrichum* sp., cinnamon essential oil inhibited more than 80% of mycelial growth at 24 h, with the inhibitory effect decreasing as the essential oil concentration was reduced. Notably, the antifungal activity of this essential oil remained relatively stable over the 120 h evaluation period at concentrations between 1000 and 500 mg/L (Figure 5A). Rosemary essential oil showed complete inhibition of mycelium growth at 1000 mg/L during the first 24 h. At lower concentrations (750, 500, and 250 mg/L), the inhibition of mycelial growth was approximately 90, 80, and 70%, respectively (Figure 5B). However, its activity declined over time, reaching around 50% inhibition after 120 h across the tested concentrations. The essential oil obtained from allspice reduced mycelial growth by approximately 80% after 120 h at concentrations ranging from 1000 to 500 mg/L, whereas inhibition decreased to about 50% at 250 mg/L. Unlike the observations for *B. cinerea*, allspice essential oil did not induce visible pigment secretion into the culture medium during the growth of *Colletotrichum* sp. Finally, Peruvian pepper essential oil exhibited minimal antifungal activity, inhibiting less than 10% of mycelial growth of *Colletotrichum* at all tested concentrations. Overall, cinnamon and allspice essential oils showed the strongest and most consistent inhibitory effects against *Colletotrichum* sp. over the five-days evaluation period.

### 3.3. Chemical Composition of Essential Oils

To better understand variations in antifungal activity, which may be linked to differences in chemical composition, the essential oils were analyzed using GC-MS. A total of 47 volatile compounds were detected across the four essential oils, including monoterpenes, sesquiterpenes, aromatic aldehydes, esters, and one fatty acid. Most essential oils displayed distinctive species-specific chromatographic profiles characterized by at least one dominant peak. Only allspice essential oil showed two major peaks corresponding to eugenol and methyleugenol (Supplementary Figure S1).

Among the analyzed oils, cinnamon essential oil contained the highest number of identified volatile compounds (29), followed by rosemary, allspice, and Peruvian pepper with 23, 19, and 17 compounds, respectively. The chemical composition of cinnamon essential oil was dominated by monoterpenoids (69%), followed by aldehydes (10%), sesquiterpenoids (7%), esters (7%), phenolic (3%), and aromatic compounds (3%). Rosemary essential oil consisted primarly of monoterpenoids (87%), along with a smaller content of sesquiterpenoids (9%), and esters (4%). Similarly, allspice essential oil contained mainly monoterpenoids (74%), and less content of sesquiterpenoids (16%), and phenolic compounds (10%). In contrast, the essential oil of Peruvian pepper was more equilibrated between the ratio of monoterpenoids (47%) and sesquiterpenoids (41%), with a smaller content of aldehydes (12%).

The essential oils exhibiting the strongest antifungal activity were generraly characterized by higher proportions of monoterpenoid compounds. However, in cinnamon essential oil, the major constituent was (*E*)-cinnamaldehyde, a phenylpropanoid. Other abundant compounds in cinnamon oil were cinnamyl acetate, sabinene, and (*E*)-*β*-caryophyllene. In rosemary essential oil, the predominat constituents were (+)-2-bornanone, *β*-myrcene, and eucalyptol. The main constituents of allspice essential oil were eugenol, methyl eugenol, (*Z*)-*β*-ocimene, eucalyptol, and *β*-myrcene. Finally, the principal compounds iddentified in Peruvian pepper essential oil were *α*-phellandrene, *α*-pinene, *β*-myrcene, *o*-cymene, and *β*-terpinene. Some compounds were detected in more than one essential oil. For example, *β*-myrcene, was present in several samples but was more abundant in Peruvian pepper essential oil, whereas eucalyptol was more abundant in rosemary essential oil (Table 1).

### 3.4. In Silico Determination of Potential Antifungal Mechanisms

The 14 main volatile compounds identified in the four essential oils were analized using the PASS-software to predict their bioactivity spectra associated with antifungal effects. In total, 56 potential mechanisms related to antifungal effects were predicted. The most frequently predicted mechanisms, present in at least half of the evaluated volatiles, included inhibition of feruloyl esterase, GABA aminotransferase inhibition, glucose-phosphate inhibition, fatty acyl-CoA synthetase inhibition, linoleate diol synthase inhibition, and both inhibition and enhancement of membrane permeability (Table 2). Among these mechanisms, only feruloyl esterase inhibition is specific for fungi, whereas the remaining predicted mechanisms are related to broader antimicrobial effects. In addition to those common mechanisms, there were distinctive mechanisms predicted only for few compounds and exclusive mechanisms predicted only for one compound. For instance, mitochondrial processing peptidase inhibition was predicted for cinnamaldehyde and *o*-cymene, while sterol 3-beta-glucosyltransferase inhibition was predicted for (*E*)-cinnamaldehyde and *β*-myrcene. Similarly, pyruvate decarboxylase inhibition was predicted for (*E*)-cinnamaldehyde and cinnamyl acetate. The membrane integrity antagonist activity was predicted for *α*-phellandrene and *o*-cymene, while the peroxidase inhibitor character was predicted only for eugenol and *β*-ocimene. The inhibition of cutinase was detected only for *β*-terpinene and *β*-ocimene.

As previously mentioned, some mechanisnms were exclusively detected for individual compounds. For example, inhibition of the steroid 17-alpha-hydroxylase/17,20 lyase was predicted only for *α*-pinene, while GABA aminotransferase inhibition was uniquely predicted for eugenol. Fatty acid synthase inhibition was predicted exclusively for *β*-ocimene. In addition, inhibition of fumarate reductase (NADH), glycerol dehydratase, and ATPase were predicted only for cinnamyl acetate. Inhibition of catalase was predicted exclusively for cinnamaldehyde. Finally, *β*-myrcene was the only volatile predicted to inhibit both NADH-related enzymes and squalene synthetase (Table 2).

### 3.5. Grouping of Volatiles Based on Predicted Bioactivity Mechanism Profiles

**Figure 6:**
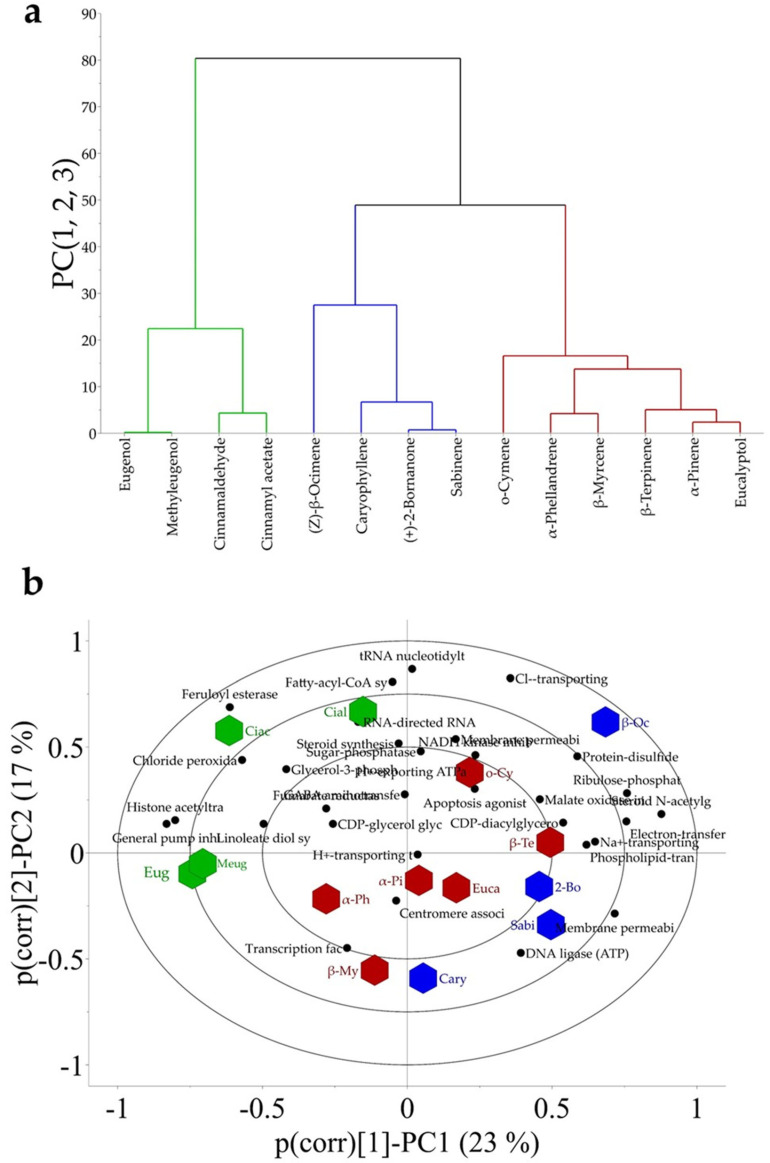
Multivariate data analysis of antifungal mechanisms predicted by the PASS-software or 14 main volatiles identified in essential oils from cinnamon, allspice, Peruvian pepper, and rosemary. (**a**) represents a hierarchical cluster analysis projected on (**b**) B-plot of a PCA model. Eug = eugenol, Meug = methyleugenol, Cial = cinnamaldehyde, Ciac = cinnamyl acetate, *α*-Ph = *α*-phellandrene, *α*-Pi = *α*-pinene, *β*-My = *β*-Myrcene, *β*-Te = *β*-terpinene, Euca = Eucalyptol, Sabi = sabinene, 2-Bo = (+)-2-bornanone, Cary = caryophyllene, and *β*-Oc = *β*-ocimene.

The prediction values of each potential antifungal mechanism predicted for all main volatiles were used to build a database for multivariate data analysis. Hierarchical cluster analysis (HCA) projected on the Bi-plot of a principal component analysis (PCA) showed three main groups of volatiles (Figure 6a). The first group included eugenol, methyleugenol, (*E*)-cinnamaldehyde, and cinnamyl acetate. The second group was formed by (*Z*)-*β*-ocimene, caryophyllene, (+)-2-bornanone and sabinene, and the third group by *o*-cymene, *α*-phellandrene, *β*-myrcene, *β*-terpinene, *α*-pinene, and eucalyptol. The PCA Bi-plot also unveiled specific associations between individual compounds and predicted mechanisms of action. For instance, inhibition of fatty-acyl-CoA and RNA polymerase was more strongly associated with (*E*)-cinnamaldehyde, whereas inhibition of the feruloyl esterase was more closely associated with cinnamyl acetate, two of the main constituents of cinnamon essential oil. Eugenol and methyl eugenol, the principal components of allspice essential oil, were associated with general efflux pump and histone acetyl transferase inhibition. The major constituent of rosemary oil, (+)-2-bornanone, was associated with membrane permeability enhancing effects; whereas *β*-myrcene was associated with inhibition of transcription factors (Figure 6b). Finally, the main volatiles of Peruvian pepper essential oil, for example *α*-phellandrene, were associated with the inhibition of transcription factors, while *β*-terpinene was associated with inhibition of CDP-glycerol glycerophosphotransferase (Figure 6b). Notably, *β*-myrcene was also detected as a major compound in Peruvian pepper essential oil.

## 4. Discussion

Among all tested plant species, the fruits of Peruvian pepper exhibited the highest essential oil yield. Previous studies have reported yields of up to 5% from fruits and approximately 2% from leaves of this species [17]. For dried fruits specifically, reported essential oil yields range between 2.8% and 4.6% [17,18]. Therefore, the yield obtained in the present study falls within the range commonly reported for this plant material. In the case of allspice, the essential oil yield obtained in this study (0.90%) was higher than the value previously reported for allspice fruits (0.38%) [19]. However, other studies have described higher yields, reaching 1.89% in fruits and 1.02% in leaves [20]. For rosemary, the essential oil yield obtained in the present study was higher than that reported for fresh plant material (0.61%) and closer to values described for dried rosemary (0.98%) [21]. Finally, the essential oil yield obtained from cinnamon in this study was lower than values previously reported in the literature, which range from 2.7% to 3.1% [22]. Overall, the essential oil yields obtained for these plant species are consistent with values previously reported in the literature. Some variations might be explained by genotype or environmental factors. The seasonal variation in rosemary essential oil content illustrates this point, with yields of 1.03% in winter and 2.23% in spring [13]. Such variability in essential oil yield has also been reported for other aromatic plant species, including thyme (*Thymus vulgaris*), basil (*Ocimum basilicum*), and oregano (*Origanum vulgare*) [13,23,24]. Therefore, considering its relatively high essential oil yield, Peruvian pepper fruits initially appeared to be the most promising plant material for further evaluation in fungal management strategies.

However, the antifungal efficacy of each essential oil must be considered alongside its extraction yield when considered as a potential fungal management agent. In the present study, despite its high yield, Peruvian pepper essential oil did not exhibit inhibitory activity against *B. cinerea* or *Colletotrichum* sp. In contrast, cinnamon and rosemary essential oils demonstrated the greatest potential for managing these postharvest fungi. For cinnamon essential oil, a concentration of 750 mg/L was sufficient to completely inhibit the growth of *B. cinerea*, which was lower than the concentration reported in a previous study to fully inhibit the same fungal species (850 mg/L). This slight difference may be attributed to differences in the experimental design, as the previous study employed a diffusion test, which may limit the movement of volatiles through the aqueous culture medium [9]. Similarly, rosemary essential oil achieved the complete inhibition of *B. cinerea* at a concentration of 750 mg/L, indicating a comparable antifungal potency against this phytopatogen. However, compared with rosemary oil, which achieved only 31.9% mycelial growth reduction at 2000 mg/L in the culture medium, our rosemary oil was two times more bioactive [25]. Nonetheless, another study on vapor phase determined 363.64 mg/L as the minimum inhibitory concentration for the same oil type [10].

The antifungal activity of allspice essential oil has been evaluated against several fungal species, although its activity against *Botrytis cinerea* has been less extensively documented. For example, minimum inhibitory concentration (MIC) values of 0.16 and 0.43 mg/mL have been reported against *Aspergillus ochraceus* and *Fusarium moniliforme*, respectively [26]. Regarding Peruvian pepper, previous studies have reported limited antifungal activity against *B. cinerea*, with approximately 24.7% inhibition observed at 500 mg/L when the essential oil was incorporated into potato dextrose agar (PDA) medium [27]. In the present study, Peruvian pepper essential oil at the same concentration inhibited approximately 70% of *B. cinerea* mycelial growth after 24 h. However, this inhibitory effect decreased to approximately 25% after five days of incubation. The decline in antifungal activity over time was dose-dependent, suggesting a predominantly fungistatic effect against *B. cinerea* at the evaluated concentrations.

Regarding antifungal activity against *Colletotrichum* sp., rosemary essential oil showed the strongest inhibitory effect during the first 24 h of the assay. However, its activity was reduced by more than 50% across all tested concentrations over time. This observation is consistent with previous reports describing complete inhibition of *Colletotrichum gloeosporioides* when 300 mg/L of rosemary essential oil was incorporated into the culture medium, with reduced inhibition observed at lower concentrations [28]. In contrast, although cinnamon essential oil did not completely inhibit the growth of *Colletotrichum* sp., its fungistatic effect remained relatively stable throughout the entire bioassay period. A separate study demonstrated that the vapor phase achieved 100% inhibition of *Colletotrichum acutatum* spore germination using cinnamon oil at concentrations of 200 and 225 mg/L [29]. Another study reported reductions of approximately 50% and 40% in the mycelial growth of *Colletotrichum gloeosporioides* and *C. acutatum*, respectively, when 2% cinnamon essential oil was incorporated into the culture medium [30]. Moreover, the fungicidal effect of cinnamon essential oil was reported at 0.1% against both *C. gloeosporioides* and *C. acutatum* when the oil was mixed directly into the culture medium.

Regarding the antifungal activity of allspice essential oil, the incorporation of 0.1% (*v*/*v*) into PDA medium produced approximately 89% inhibition of *C. gloeosporioides* growth [31]. However, the antifungal efficacy of this oil has been shown to be concentration-dependent, with strong inhibitory effects reported at concentrations close to 2 mg/mL [32]. In contrast, Peruvian pepper essential oil exhibited only mild antifungal activity across the tested concentrations. This observation is consistent with previous reports describing approximately 15% inhibition of *C. gloeosporioides* growth at 160 mg/L of Peruvian pepper essential oil incorporated into the culture medium [33].

The variability in antifungal activity among essential oils against *B. cinerea*, *Colletotrichum* sp., and other fungi reported in the literature may partly reflect differences among fungal species. Variations in cell wall composition, membrane structure, and defense mechanisms can influence fungal susceptibility to plant-derived volatiles, ultimately determining the final outcome of fungal–volatile interactions [34,35]. However, the GC–MS analysis conducted in our study suggests that differences in antifungal activity may also be associated with variations in essential oil chemical composition.

In this context, the chemical analysis revealed notable differences in the abundance of compounds previously reported to possess antifungal activity. For example, (*E*)-cinnamaldehyde, the major constituent of cinnamon essential oil, has been widely reported to inhibit several postharvest pathogens, including *B. cinerea*, *Penicillium expansum*, and *Penicillium italicum*. Its antifungal activity is largely attributed to the presence of the reactive aldehyde functional group, which can covalently interact with amino groups in proteins and nucleic acids, thereby interfering with their normal biological functions. (*E*)-cinnamaldehyde has been reported to exert antifungal activity at concentrations lower than those required for whole cinnamon essential oil [36,37,38].

Similarly, eugenol, the principal component of allspice essential oil, has been widely described as a potent antifungal metabolite. The mode of action of eugenol involves interaction with the fungal cell membrane, leading to reduced ergosterol synthesis and subsequent disruption of membrane permeability [39,40,41]. The membrane alterations can result in cellular leakage and morphological changes in fungal hyphae, including cytoplasmic coagulation, vacuolation, and hyphal shrinkage [41]. In *C. gloeosporioides*, eugenol has also been reported to inhibit mycelial growth, interfere with appressorium formation, and reduce conidial germination [42].

In the case of (+)-2-bornanone, the major component of rosemary essential oil, antifungal activity has been associated with membrane-disruptive effects. Such disruption increases membrane permeability, leading to the leakage of intracellular proteins and nucleic acids [43]. Interestingly, although *α*-phellandrene, an antifungal monoterpene, was the most abundant metabolite in Peruvian pepper essential oil, this oil showed minimal inhibitory activity against *B. cinerea* and *Colletotrichum* sp. Previous studies have shown that *α*-phellandrene can affect the membrane integrity; however, concentrations of at least 400 mg/L were required to completely inhibit the growth of *Penicillium cyclopium* [44]. Therefore, the relatively low antifungal activity observed in this study may be related to insufficient concentrations of this metabolite within the oil mixture. Nonetheless, another possible explanation for the variability in antifungal activity among essential oils involves metabolite interactions, including synergistic and antagonistic effects. For example, cinnamyl acetate has been reported to exhibit lower antifungal activity than (*E*)-cinnamaldehyde but can enhance the antifungal effect of (*E*)-cinnamaldehyde when mixed [45]. In contrast, *β*-myrcene, detected in all analyzed essential oils, has been described as a relatively weak antifungal compound despite its ability to disrupt cell membranes, induce oxidative stress and apoptosis, and alter lipid dynamics and fungal gene expression [46,47].

Interestingly, *β*-myrcene has also been reported to antagonize the activity of stronger antimicrobial compounds. For example, while *α*-pinene, camphene, *α*-terpinene, and *p*-cymene enhance the antibacterial activity of carvacrol, *β*-myrcene reduces its efficacy [45]. This antagonistic effect has been attributed to the capacity of *β*-myrcene to reduce the aqueous solubility and bioavailability of carvacrol [45,48]. In the present study, a similar antagonistic interaction between *α*-phellandrene and *β*-myrcene could explain the limited antifungal activity of Peruvian pepper essential oil. Moreover, the presence of *β*-myrcene in active oils such as rosemary and allspice suggests that its influence may depend on concentration or on specific structural interactions with other metabolites, highlighting the importance of metabolite composition and synergistic interactions in determining the overall antifungal activity of essential oils [29].

Finally, another factor influencing antagonist or synergistic interactions among metabolites is the compatibility of their modes of action, which may interact either negatively or positively when combined [49,50]. Therefore, understanding the mechanistic spectrum of individual volatile compounds could facilitate the selection of metabolites with a greater likelihood of producing synergistic antifungal effects. Such knowledge may contribute to the rational design of volatile formulations with potential applications in the management of pathogenic fungi [45]. However, information about specific mechanisms of several volatiles remains limited. In this context, the in silico analysis of the major volatiles of each evaluated essential oil provided a broader overview of their biological potential. The analysis predicted 56 mechanisms associated with general antimicrobial or specific antifungal activity. The antifungal mechanisms can be generally classified according to their functional targets.

One group of mechanisms includes interference with lipid and membrane biosynthesis. These mechanisms involve the inhibition of enzymes such as acyl-CoA synthetase, phospholipid-translocase ATPase, linoleoyl-CoA desaturase, fatty acid synthase, feruloyl esterase, and squalene synthase. Additionally, inhibition of steroid hydroxylases, glucuronosyltransferases, enzymes involved in the mevalonate pathway, and other enzymes associated with sterol biosynthesis were predicted. In eukaryotic cells, these metabolic pathways are primarily located in the endoplasmic reticulum, and they are essential for maintaining membrane integrity and cellular homeostasis [51]. A second group of mechanisms includes those predicted to act as cellular transport inhibitors. These mechanisms interfere with ion transport systems and proton pumps such as H^+^-, Na^+^-, and Cl^−^-ATPases, as well as dual-sector ion pumps. By disrupting ionic homeostasis, these compounds may alter electrochemical gradients across the plasma membrane and mitochondrial membranes, processes that are critical for maintaining cell viability [52].

A third group of mechanisms includes compounds capable of inducing oxidative stress by targeting mitochondrial and cytoplasmic antioxidant systems. These mechanisms involve the inhibition of enzymes such as catalases, peroxidases, oxidoreductases, and glutathione reductase, which can lead to the accumulation of reactive oxygen species (ROS) and subsequent cellular damage [53]. The fourth group comprises mechanisms affecting primary energy metabolism. These include the inhibition of mitochondrial enzymes such as glycerol-3-phosphate dehydrogenase, fumarate reductase, malate oxidase, and pyruvate decarboxylase, ultimately reducing cellular energy production and compromising fungal viability [54]. A fifth group includes mechanisms targeting nucleic acid processes during replication, transcription, or translation. These mechanisms involve the inhibition of enzymes and regulatory proteins such as RNA polymerase, RNA nucleotidyltransferase, DNA ligases, histone acetyltransferases, and transcription factors.

Finally, a sixth group includes mechanisms that affect critical cellular processes at the protein level, such as inhibition of mitochondrial protein synthesis, disruption of centromere- and cytoskeleton-associated proteins, alteration of intracellular pump regulation, and activation of pro-apoptotic pathways. Considering the mechanistic classification, several of the predicted mechanisms were associated with more than one volatile compound and exhibited different prediction probabilities. Such complexity makes it difficult to identify the most promising antifungal volatiles based solely on individual mechanistic predictions. Therefore, multivariate modeling was applied to simplify the interpretation of the mechanistic profiles. The analysis grouped the 14 major volatiles into three main clusters according to their predicted mechanisms of action. Interestingly, (*E*)-cinnamaldehyde, cinnamyl acetate, eugenol, and methyl eugenol formed the most distinct cluster in the hierarchical analysis. This separation may be related to their structural similarity, as these compounds are phenylpropanoid derivatives that likely share related antifungal modes of action.

Two additional clusters were also identified. The second cluster included (*Z*)-*β*-ocimene, (*E*)-*β*-caryophyllene, (+)-2-bornanone, and sabinene, whereas the third cluster comprised *o*-cymene, α-phellandrene, *β*-myrcene, *β*-terpinene, *α*-pinene, and eucalyptol. Notably, the first cluster contained compounds with the strongest antifungal activities reported in the literature, whereas the remaining clusters were primarily composed of terpenes that may act as activity modulators or enhancers [45]. To identify the mechanisms most strongly associated with each volatile compound, a PCA biplot was constructed. The analysis revealed that (*E*)-cinnamaldehyde and cinnamyl acetate were characterized by higher probabilities of inhibiting fatty acyl-CoA synthetase, RNA polymerase, and feruloyl esterase. The latter mechanism is particularly noteworthy because it may not directly inhibit fungal growth but rather interfere with fungal pathogenicity. Feruloyl esterases catalyze the hydrolysis of ester bonds between ferulic acid and plant cell wall polysaccharides, functioning as auxiliary enzymes that assist xylanolytic enzymes and pectinases in accessing their substrates. By facilitating the degradation of plant cell wall components, these enzymes contribute to the pathogen penetration and colonization of plant tissues [55].

Eugenol and methyl eugenol were distinguished by their high predicted probability of inhibiting general efflux pump systems and histone acetyltransferases (HATs). In fungi, HATs play a critical role in regulating gene expression by catalyzing the acetylation of histone proteins. This modification influences key fungal processes, including growth, sporulation, toxin production, and virulence [56]. These findings suggest that essential oil volatiles may possess biological activities extending beyond simple membrane-disrupting effects. From a bioinspiration perspective, the multivariate interpretation of the in silico data indicates that (*E*)-cinnamaldehyde, cinnamyl acetate, eugenol, and methyl eugenol could serve as a foundational mixture for the development of antifungal formulations. This base formulation could potentially be enhanced by incorporating additional volatiles from the other two identified clusters, thereby increasing the diversity of antifungal mechanisms within the mixture. An initial strategy could involve testing the most distinct compounds from each cluster, such as (*Z*)-*β*-ocimene and *o*-cymene, followed by the sequential inclusion of volatiles from the larger clusters. Interestingly, (*Z*)-*β*-ocimene and *β*-terpinene were the only two volatiles predicted to inhibit cutinase activity. Cutinase is an important enzyme involved in early plant–fungus interactions, as it catalyzes the degradation of cutin, a fatty acid polyester that constitutes the main structural component of the plant cuticle [57,58]. By facilitating the degradation of this barrier, fungal pathogens can initiate host tissue colonization. Therefore, inhibition of cutinase may represent an effective strategy to interfere with early stages of fungal infection.

Similarly, *o*-cymene was one of the few volatiles predicted to inhibit catalase activity. Catalase plays a crucial role in fungal defense against oxidative stress generated during plant–pathogen interactions. Plants often produce reactive oxygen species (ROS) as part of their defense response, and fungal catalases help neutralize these radicals to protect cellular integrity [53]. Consequently, inhibition of catalase could weaken fungal resistance to plant-derived oxidative stress. Furthermore, both (*Z*)-*β*-ocimene and *o*-cymene were predicted to inhibit linoleoyl-CoA desaturase, an enzyme involved in lipid metabolism and membrane homeostasis. In addition, *o*-cymene was uniquely predicted to exhibit sodium channel blocker class Ib activity, whereas (*Z*)-*β*-ocimene showed predicted inhibition of mevalonate kinase and fatty acid synthase. These targets are closely associated with fungal cellular homeostasis, membrane integrity, and metabolic regulation [52,54]. Overall, these predictions highlight the potential of combining volatile metabolites with complementary mechanisms of action to develop multifunctional antifungal formulations. Nevertheless, confirmatory in vitro and in vivo experiments will be necessary to validate these predicted mechanisms and to optimize synthetic formulations inspired by the biological potential of essential oil metabolites for the control of postharvest fungal pathogens.

## 5. Conclusions

In this study, the antifungal activity of four essential oils was evaluated against the postharvest phytopatogens *Botrytis cinerea* and *Colletotrichum* sp. Among the tested oils, cinnamon and rosemary exhibited the strongest inhibitory effects against both fungal species. Allspice essential oil showed moderate antifungal activity, whereas Peruvian pepper essential oil displayed the lowest inhibitory effect. The Chemical characterization revealed the presence of several bioactive metabolites, among which cinnamaldehyde, cinnamyl acetate, eugenol, methyl eugenol, (+)-2-bornanone, eucalyptol, *α*-phellandrene, and *β*-myrcene were particularly abundant and have been previously associated with antifungal activity. The in silico analyses predicted multiple potential mechanisms of antifungal action, including inhibition of cell membrane and cell wall biosynthesis, disruption of structural integrity, interference with primary metabolic pathways, inhibition of nucleic acid processes (replication, transcription, and translation), perturbation of redox homeostasis, and effects on protein metabolism. The integration of in silico prediction with multivariate data analysis provided a comprehensive framework for interpreting the antifungal potential of volatile metabolites and represents a promising strategy for the rational design of bioinspired antifungal formulations. Particularly, compounds with predicted cutinase inhibition activity, such as *β*-terpinene and *β*-ocimene, may complement the activity of other bioactive metabolites that lack this mechanism. Based on the literature and the in silico predictions obtained in this study, a mixture containing cinnamaldehyde, eugenol, *β*-terpinene, and *β*-ocimene could represent a promising candidate formulation for the management of postharvest fungal pathogens. However, further in vivo studies are required to validate the efficacy of such formulations and to determine optimal qualitative and quantitative combinations of volatiles. Ultimately, these findings may contribute to the development of synthetic formulations inspired by essential oil metabolites, potentially reducing dependence on natural essential oil sources for antifungal applications.

## Figures and Tables

**Table 1:** Chemical composition of essential oils obtained from Cinnamon (*Cinnamomum verum*), allspice (*Pimienta dioica*), Peruvian pepper (*Schinus molle*), and rosemary (*Salvia Rosmarinus*) analyzed by GC-MS.

Compound	Score Match (%)	*C. verum*	*P. dioica*	*S. molle*	*S. rosmarinus*
Monoterpenoids					
Tricylene	93	nd	nd	nd	0.27 ± 0.09
*β*-Thujene	93	0.46 ± 0.06	nd	nd	nd
*α*-Thujene	95	nd	nd	nd	0.28 ± 0.02
*α*-Pinene *	96	1.82 ± 0.16	0.16 ± 0.01	4.07 ± 0.05	11.66 ± 1.09
Camphene *	97	0.86 ± 0.08	nd	nd	4.79 ± 0.53
Sabinene isomer	95	0.08 ± 0.02	0.31 ± 0.01	0.12 ± 0.00	nd
*β*-Pinene *	97	0.61 ± 0.06	0.17 ± 0.01	0.18 ± 0.01	5.23 ± 0.24
*β*-Myrcene *	95	0.28 ± 0.04	19.41 ± 0.37	25.81 ± 0.70	12.45 ± 0.46
*α*-Phellandrene *	91	2.75 ± 0.33	nd	33.56 ± 0.39	1.88 ± 0.19
3-Carene *	95	0.12 ± 0.01	nd	nd	nd
Terpinolene	97	2.73 ± 0.30	nd	nd	nd
*α*-Terpinene	96	nd	0.17 ± 0.00	nd	0.63 ± 0.02
*o*-Cymene *	97	2.09 ± 0.17	nd	6.06 ± 0.31	0.53 ± 0.02
*β*-Terpinene	81	nd	nd	24.18 ± 0.26	5.84 ± 0.10
Sabinene isomer	94	9.85 ± 1.02	nd	nd	nd
D-Limonene *	98	nd	0.57 ± 0.01	nd	nd
Eucalyptol *	99	0.36 ± 0.02	3.15 ± 0.04	0.37 ± 0.08	16.43 ± 0.86
(*E*)-*β*-ocimene *	97	0.16 ± 0.02	nd	nd	nd
(*Z*)-*β*-ocimene *	98	0.17 ± 0.03	3.45 ± 0.12	nd	nd
*γ*-Terpinene *	96	0.36 ± 0.04	0.23 ± 0.01	nd	1.54 ± 0.02
Terpinolene	98	0.20 ± 0.07	0.41 ± 0.09	0.12 ± 0.01	0.56 ± 0.02
Cyclofenchene	89	nd	0.57 ± 0.01	nd	0.55 ± 0.03
(+)-2-Bornanone *	98	nd	nd	nd	26.15 ± 0.52
*endo*-Borneol	97	nd	nd	nd	2.60 ± 0.05
Pinocamphone	96	nd	nd	nd	0.27 ± 0.02
*trans*-Verbenone	96	nd	nd	nd	0.22 ± 0.06
Terpinen-4-ol	97	1.12 ± 0.12	0.56 ± 0.00	nd	1.27 ± 0.08
*α*-Terpineol *	91	0.82 ± 0.05	0.44 ± 0.01	nd	1.40 ± 0.21
Copaene	99	0.31 ± 0.04	0.06 ± 0.02	nd	nd
*α*-Gurjunene	86	nd	nd	0.22 ± 0.03	nd
Bornyl acetate	99	nd	nd	nd	3.69 ± 0.07
Sesquiterpenoids					
(*E*)-*β*-caryophyllene *	99	5.41 ± 0.56	1.89 ± 0.55	0.47 ± 0.12	0.64 ± 0.05
Humulene	96	1.03 ± 0.08	0.50 ± 0.02	0.13 ± 0.02	0.17 ± 0.02
Germacrene D	97	nd	0.24 ± 0.01	0.34 ± 0.03	nd
Bicyclogermacrene	98	nd	nd	0.45 ± 0.15	nd
*γ*-muurolene	90	nd	nd	0.12 ± 0.05	nd
*δ*-cadinene	96	nd	nd	0.73 ± 0.18	nd
Benzene derivatives					
Benzaldehyde *	97	0.35 ± 0.02	nd	nd	nd
Propanalbenzene	98	0.69 ± 0.05	nd	nd	nd
(*Z*)-3-Phenylacrilaldehyde	98	0.76 ± 0.26	nd	nd	nd
(*E*)-Cynnamaldehyde *	98	49.03 ± 2.70	nd	nd	nd
Eugenol *	98	2.49 ± 0.55	34.67 ± 0.36	nd	nd
Methyleugenol *	99	nd	31.60 ± 0.64	nd	nd
3-Phenylpropil formiate	91	0.29 ± 0.03	nd	nd	nd
Cinnamyl acetate *	97	7.69 ± 0.38	nd	nd	nd
Benzylbenzoate	98	0.25 ± 0.02	nd	nd	nd
Fatty acid					
Octanoic acid	90	nd	nd	1.24 ± 0.08	nd

The composition % percentage was calculated from the sum of total peak areas in each chromatogram. Values in the table represent percentage composition of each compound ± standard error. * Compound identified by standard compounds’ retention time and mass spectrum comparison; nd = no detected.

**Table 2:** Potential mechanisms related to the antifungal activity of major volatiles from the essential oils of cinnamon (*Cinnamomum verum*), allspice (*Pimienta dioica*), Peruvian pepper (*Schinus molle*) and rosemary (*Salvia rosmarinus*).

Potential Mechanism of Action	*α*-Pi	*α*-Ph	Eugl	Meug	Cial	Ciac	*β*-My	Eucl	Born	*β*-Te	Cary	*o*-cy	Sabi	*β*-oc
Sugar-phosphatase inhibitor	0.611	nd	0.513	0.528	0.758	0.791	nd	nd	0.577	0.663	0.433	nd	0.545	0.777
Fatty-acyl-CoA synthase inhibitor	0.55	0.612	0.714	0.70	0.758	0.69	nd	0.513	0.513	0.589	0.363	0.764	0.438	0.929
Steroid N-acetylglucosaminyltransferase inhibitor	0.481	nd	nd	nd	0.424	nd	nd	0.505	0.567	0.453	0.457	0.535	0.515	0.695
Phospholipid-translocating ATPase inhibitor	0.452	nd	nd	nd	nd	nd	nd	0.448	0.386	nd	nd	nd	0.353	0.581
Chloride peroxidase inhibitor	0.446	0.401	nd	0.532	0.708	0.601	nd	nd	nd	nd	nd	nd	nd	nd
Membrane permeability inhibitor	0.428	0.671	nd	0.701	0.615	0.658	nd	nd	0.508	0.634	0.373	0.781	0.548	0.635
H+-exporting ATPase inhibitor	0.416	0.73	nd	nd	0.648	nd	nd	0.47	nd	0.506	nd	0.672	nd	0.305
GABA aminotransferase inhibitor	0.39	nd	nd	nd	0.604	0.563	0.508	nd	0.429	0.322	0.32	nd	0.308	0.587
Cl--transporting ATPase inhibitor	0.365	0.429	nd	nd	0.596	0.645	nd	0.408	0.387	0.558	nd	0.684	0.361	0.776
Oxidizing agent	0.344	nd	nd	nd	nd	nd	nd	nd	nd	nd	nd	nd	nd	nd
Steroid 17-alpha-hydroxylase/17,20 lyase inhibitor	0.342	nd	nd	nd	nd	nd	nd	nd	nd	nd	nd	nd	nd	nd
NADH kinase inhibitor	0.342	nd	nd	nd	nd	nd	nd	nd	nd	nd	nd	nd	nd	nd
Na+-transporting two-sector ATPase inhibitor	0.341	nd	nd	nd	nd	nd	nd	nd	nd	nd	nd	0.54	nd	0.392
Feruloyl esterase inhibitor	0.334	0.426	0.876	0.831	0.953	0.914	0.489	0.343	nd	0.404	nd	0.764	nd	0.611
Centromere associated protein inhibitor	0.321	0.402	nd	nd	nd	nd	nd	0.381	nd	nd	nd	nd	nd	nd
Linoleate diol synthase inhibitor	nd	0.56	0.859	0.707	nd	0.483	0.368	0.32	nd	nd	nd	0.726	0.37	0.472
CDP-glycerol glycerophosphotransferase inhibitor	nd	0.328	0.668	0.64	nd	nd	nd	0.44	nd	nd	nd	nd	nd	0.742
GABA aminotransferase inhibitor	nd	nd	0.619	nd	nd	nd	nd	nd	nd	nd	nd	nd	nd	nd
General pump inhibitor	nd	0.559	0.608	0.564	nd	0.733	nd	nd	nd	nd	nd	nd	nd	nd
Glycerol-3-phosphate dehydrogenase inhibitor	nd	nd	0.559	0.48	0.48	nd	nd	nd	nd	nd	nd	nd	nd	0.379
Peroxidase inhibitor	nd	nd	0.548	nd	nd	nd	nd	nd	nd	nd	nd	0.472	nd	nd
Histone acetyltransferase inhibitor	nd	nd	0.449	0.333	nd	0.336	nd	nd	nd	nd	nd	nd	nd	nd
Transcription factor inhibitor	nd	nd	0.387	0.348	nd	nd	nd	nd	nd	nd	0.417	nd	0.523	nd
Cl--transporting ATPase inhibitor	nd	nd	0.327	nd	nd	nd	nd	nd	nd	nd	nd	nd	nd	nd
H+-transporting two-sector ATPase inhibitor	nd	0.717	nd	nd	nd	nd	nd	nd	nd	0.355	nd	0.334	nd	nd
Membrane integrity antagonist	nd	0.642	nd	nd	nd	nd	nd	nd	nd	nd	nd	0.712	nd	nd
Membrane permeability enhancer	nd	0.499	nd	nd	nd	nd	0.486	0.379	0.415	0.467	0.359	0.516	0.481	0.49
Apoptosis agonist	nd	nd	nd	0.713	0.906	0.42	nd	nd	0.472	0.63	0.867	nd	0.538	0.853
Glycerol-3-phosphate oxidase inhibitor	nd	nd	nd	0.373	nd	nd	nd	nd	nd	nd	nd	nd	nd	nd
Sterol 3-beta-glucosyltransferase inhibitor	nd	nd	nd	nd	0.341	nd	0.314	nd	nd	nd	nd	nd	nd	nd
RNA-directed RNA polymerase inhibitor	nd	nd	nd	nd	0.396	0.407	nd	0.329	nd	nd	nd	0.355	nd	nd
Mitochondrial intermediate peptidase inhibitor	0.423	nd	nd	nd	nd	0.405	nd	nd	nd	nd	nd	nd	nd	nd
Steroid synthesis inhibitor	nd	nd	nd	nd	0.424	0.45	nd	0.429	0.338	nd	nd	0.363	nd	nd
Mitochondrial processing peptidase inhibitor	nd	nd	nd	nd	0.432	nd	nd	nd	nd	nd	nd	0.32	nd	nd
tRNA nucleotidyltransferase inhibitor	nd	nd	nd	nd	0.433	0.398	nd	nd	nd	nd	nd	0.485	nd	0.33
Catalase inhibitor	nd	nd	nd	nd	0.442	nd	nd	nd	nd	nd	nd	nd	nd	nd
Pyruvate decarboxylase inhibitor	nd	nd	nd	nd	0.656	0.718	nd	nd	nd	nd	nd	nd	nd	nd
Protein-disulfide reductase (glutathione) inhibitor	nd	nd	nd	nd	0.774	nd	nd	nd	0.434	0.485	nd	nd	0.525	0.719
Fumarate reductase (NADH) inhibitor	nd	nd	nd	nd	nd	0.588	nd	nd	nd	nd	nd	0.339	nd	nd
Glycerol dehydratase inhibitor	nd	nd	nd	nd	nd	0.432	nd	nd	nd	nd	nd	nd	nd	nd
ATPase inhibitor	nd	nd	nd	nd	nd	0.317	nd	nd	nd	nd	nd	nd	nd	nd
Fumarate reductase (NADH) inhibitor	nd	nd	nd	nd	nd	nd	0.443	nd	nd	nd	nd	nd	nd	nd
Squalene synthetase inhibitor	nd	nd	nd	nd	nd	nd	0.437	nd	nd	nd	nd	nd	nd	nd
Malate oxidase inhibitor	nd	nd	nd	nd	nd	nd	0.34	nd	nd	0.327	nd	0.433	nd	0.396
Na+-transporting two-sector ATPase inhibitor	nd	nd	nd	nd	nd	nd	0.316	0.411	nd	0.471	nd	nd	0.371	nd
CDP-diacylglycerol-glycerol-3-phosphate 3-phosphatidyltransferase inhibitor	nd	nd	nd	nd	nd	nd	nd	0.351	nd	nd	nd	nd	0.3	0.457
DNA ligase (ATP) inhibitor	nd	nd	nd	nd	nd	nd	nd	nd	0.367	nd	0.345	nd	0.326	nd
Electron-transferring-flavoprotein dehydrogenase inhibitor	nd	nd	nd	nd	nd	nd	nd	nd	0.499	0.625	nd	nd	0.475	0.687
NADH kinase inhibitor	nd	nd	nd	nd	nd	nd	nd	nd	0.363	nd	nd	nd	nd	0.695
Ribulose-phosphate 3-epimerase inhibitor	nd	nd	nd	nd	nd	nd	nd	nd	0.549	0.668	nd	0.772	0.525	0.69
Cutinase inhibitor	nd	nd	nd	nd	nd	nd	nd	nd	nd	0.411	nd	nd	nd	0.565
Linoleoyl-CoA desaturase inhibitor	nd	nd	nd	nd	nd	nd	nd	nd	nd	nd	nd	0.446	nd	0.358
Sodium channel blocker class Ib	nd	nd	nd	nd	nd	nd	nd	nd	nd	nd	nd	0.479	nd	nd
Oxidoreductase inhibitor	nd	nd	nd	nd	nd	nd	nd	nd	nd	nd	nd	nd	0.392	0.631
Mevalonate kinase inhibitor	nd	nd	nd	nd	nd	nd	nd	nd	nd	nd	nd	nd	nd	0.437
Fatty acid synthase inhibitor	nd	nd	nd	nd	nd	nd	nd	nd	nd	nd	nd	nd	nd	0.336

Values represent the difference between Pa and Pi in the PASS^®^ program. *α*-Pi, *α*-pinene; α-Ph, *α*-phellandrene; Eugl, eugenol; Meug, methyleugenol; Cial, cinnamaldehyde; Ciac, cinnamyl acetate; *β*-My, *β*-myrcene; Eucl, eucalyptol; Born, 2-(+)-bornanone; *β*-Te, *β*-terpinene; Cary, caryophyllene; o-cy, o-cymene; Sabi, sabinene; β-oc, β-ocimene; “nd” = no detected.

## Data Availability

Main data is fully displayed in the manuscript, and any other data is available upon request to the corresponding author.

## Supplementary Materials

The following supporting information can be downloaded at: https://www.mdpi.com/article/10.3390/metabo16040239/s1. Supplementary Figure S1. Chromatographic profiles of essential oils from (A) cinnamon (*Cinnamomum verum*), (B) allspice (*Pimenta dioica*), (C) rosemary (*Salvia rosmarinus*), and Peruvian pepper (*Schinus molle*) analyzed by gas chromatography–mass spectrometry. Chemical structures and names of the compounds correspond to the major chromatographic peaks.

## Abbreviations

The following abbreviations are used in this manuscript:

GC-MS	Gas chromatography-mass spectrometry
HCA	Hierarchical cluster analysis
PCA	Principal component analysis
UV	Unit variance
SMILES	Simplified Molecular Input Line Entry System
PASS	Prediction of Activity Spectra for Substances
