# Supplementary material for: In Vitro/In Silico Potential of High-Yield Essential Oils for Management of Postharvest Fungi
Source: Metabolites. 2026 Mar 31;16(4):239. doi: 10.3390/metabo16040239 (PMC13117349; doi:10.3390/metabo16040239)
Supplement: Supplementary file 1 [file metabolites-16-00239-s001.zip › metabolites-4204197-supplementary.pdf]

Supplementary Materials

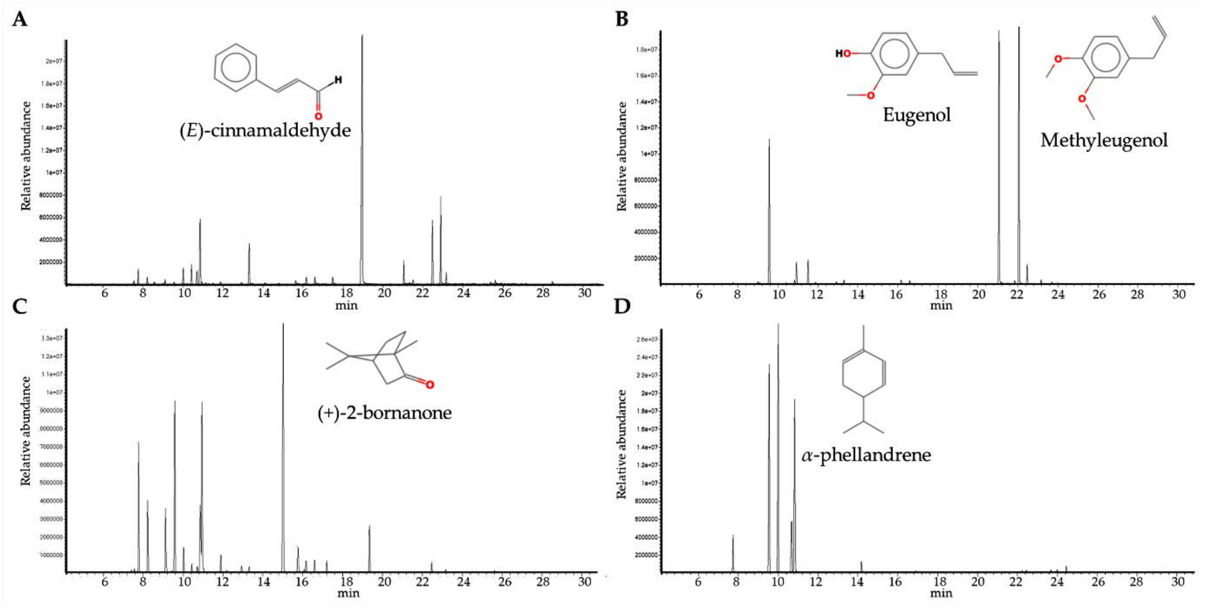

Supplementary figure S1. Chromatographic profiles of essential oils from (A) cinnamon (*Cinnamomum verum*), (B) allspice (*Pimenta dioica*), (C) rosemary (*Salvia rosmarinus*), and Peruvian pepper (*Schinus molle*) analyzed by gas chromatography–mass spectrometry. Chemical structures and names of the compounds correspond to the major chromatographic peaks.
